# Maximization strategies in relationship and career enhances life satisfaction through meaning making among established adults in South Korea

**DOI:** 10.1186/s40359-024-01672-2

**Published:** 2024-04-17

**Authors:** Yerin Shim, Yun-Jeong Shin, Ji-yeon Lee

**Affiliations:** 1 Department of Psychology, Chungnam National University, Daejeon, South Korea; 2 Department of Education, Seoul National University, Seoul, South Korea; 3 Graduate School of Education, Hankuk University of Foreign Studies, 107 Imun Ro, Seoul, 130- 791 South Korea

**Keywords:** Relational maximization, Career maximization, Maximization strategy, Life satisfaction, Search for meaning, Presence of meaning, Established adults

## Abstract

**Background:**

Choosing a partner and job have long been regarded as important developmental milestones to reach in adulthood. In a collective cultural context with high familial and societal expectations to commit to a relationship and job by age 30, maximizing on such life decisions might potentially harm one’s well-being. The literature on maximization-well-being association is complex, and recent research suggests that this relationship might differ by its dimensions and cultural context. In the present study, we examined how engaging in a maximization strategy in relationship and career domains predicts life satisfaction and whether this pathway is mediated by a meaning-making process (search-to-presence of meaning) among established adults in South Korea.

**Methods:**

Survey data on measures of relational maximization strategy, career maximization strategy, search for meaning, presence of meaning, and life satisfaction was collected from 264 South Korean unmarried, working adults in their 30 s to 40 s. A two-step structural equation modeling method was applied to test the hypothesized serial mediation model.

**Results:**

Tests of the measurement and structural model showed good fit with the data (CFI = .96, TLI = .95, RMSEA = .07, SRMR = .05). Further bootstrapping results supported significant indirect effects of the serial mediation model in the paths between maximization strategy and life satisfaction via a search-to-presence of meaning in both relational (*b* = .16, 95% CI [.01, -.16], *p* < .05) and career (*b* = .26, 95% CI [.11, -.26], *p* < .01) domains.

**Conclusions:**

Our findings suggest that for established adults in a collectivist culture who may experience familial and social pressure on these life choices, searching for alternative options to make the best relationship and career decisions could potentially lead to higher life satisfaction, when done through an intentional meaning-making process.

**Supplementary Information:**

The online version contains supplementary material available at 10.1186/s40359-024-01672-2.

Love and work have been historically regarded as two fundamental themes in an individual’s successful development by psychologists. Sigmund Freud, famously known by the quote “to love and to work (*zu lieben und zu arbeiten*),” viewed these as capacities that characterize a mature and psychologically healthy person [1]. Erik Erikson also named *intimacy* and *generativity* as major developmental milestones to be reached in adulthood [2]. Empirical research confirms that these two developmental tasks are salient for successful adult development [3], and modern psychological theories on well-being and flourishing commonly identify positive relationships and work as key indicators and pathways to a good life [4–6]. However, mixed results are found regarding whether attaining such developmental goals may or may not lead to well-being in adulthood [7, 8], suggesting that simply achieving these goals may not be a guaranteed condition for happiness.

While life choices in romantic relationships and career can provide one an opportunity for tremendous growth and positive change when done right, it could also lead to more intense regret when it goes awry [9]. Because of these two potential contrasting outcomes, decision-making in relationships and career may increase one’s tendency to maximize. *Maximization* is a personality trait that reflects the pursuit of an optimal choice by seeking out and comparing alternatives in decision-making contexts [10]. While some people are happy with a “good enough” option, maximizers tend to exhaustively search for, and examine alternative options with the goal of choosing “the best” [11]. Despite this effort to make the best decision, however, high maximization tendency has been found to be associated with detrimental psychological effects which is referred to as the “maximization paradox” [12]. This paradox has led researchers to extensively study the relationship between maximization and well-being. Moreover, according to a recent study which investigated maximization across different decision domains, deciding on a partner and job were among the few domains in which people generally showed the highest maximization tendencies [13], which may have important implications for the well-being of adults who are expected to have made these decisions by their age.

## Dimensions of maximization and well-being

The body of literature on maximization and well-being is quite complex, with mixed findings based on the dimension of maximization and well-being. Regarding maximization dimensions, a two-component model of maximization suggests that the maximization *goal* of choosing the best and the maximization *strategy* of searching alternatives should be examined separately as they tend to have opposing psychological experiences [10, 14]. A recent meta-analysis that examined the relationship between maximization dimensions and well-being further confirm that maximization *strategy* (i.e., alternative search) has a stronger and negative relationship with overall well-being than maximization *goal* (i.e., choosing the best) which had a weaker and even positive association with overall well-being [15]. Thus, maximization strategy appears to be the active ingredient in the maximization paradox.

Maximization also tends to show different relationships with various indicators of well-being. Two traditions of well-being (i.e., hedonic and eudaimonic well-being) are widely cited in the literature [5, 16] and the maximization-well-being association has also been primarily studied within this framework. For example, in the abovementioned meta-analytic study, general maximization showed stronger detrimental effects on hedonic indicators of well-being such as life satisfaction than eudaimonic indicators of well-being such as meaning in life [15]. However, previous research which examined the relationship between maximization and well-being has either measured maximization as a whole without differentiating maximization goal and strategy (e.g., [17, 18]), or have only measured only one type of well-being (either hedonic or eudaimonic) (e.g., [19]), which makes it difficult to know the specific relationship between maximization dimensions and hedonic/eudaimonic indicators of well-being. In our study, we focus on the dimension of maximization strategy and its relationship between two representative indicators of hedonic and eudaimonic well-being, life satisfaction and meaning in life, respectively.

### Maximization and life satisfaction in relationships and career decision-making

The current research synthesis on the relationship between maximization and well-being has mostly focused on decision-making in consumer behavior, and research on maximization in specific life domains such as career and romantic relationships are relatively in its nascent stage. Existing research on relational maximization has found that people with higher relational maximization tendency tend to be less satisfied with their romantic relationship [20] and are likely to get married later than those with lower maximizing tendencies [21]. Maximization strategy in relationship decision-making has also been found to be negatively correlated with life satisfaction, while maximization goal had the opposite pattern [22], supporting previous findings in general maximization research. Several studies have also explored the negative consequences of maximization in a career context. General maximization was found to be associated with negative career thoughts [23], and negative vocational consequences for both colleges students and working adults [24]. However, no study to date has directly examined the relationship between career maximization and life satisfaction, which warrants more research to clarify this relationship.

### The role of meaning-making in maximization

Based on these initial studies on maximization and life satisfaction in relationship and career domains, we can speculate that people who use maximization strategies in relationship and career-related decision-making may be vulnerable to sacrificing their life satisfaction. This warrants a need for an investigation on specific psychological routes that could enable those individuals to maintain their life satisfaction while thoughtfully engaging in the decision-making process. One such hypothesized route is through a meaning-making process through an intentional search for meaning, which leads to a presence of meaning.

While the *presence of* meaning has been a robust, positive predictor of life satisfaction across many cultures [25], the *search for* meaning tend to show different patterns in its relationship with life satisfaction depending on the cultural context. According to a meta-analytic study based on 147 studies from countries that varied in their individualism level, individualism significantly moderated the relationship between search for meaning and subjective well-being (which includes life satisfaction) but not with presence of meaning [26]. That is, the more collectivistic the culture, search for meaning played a significant role in enhancing one’s satisfaction with life. This pattern has also replicated in empirical research with Korean adults, which consistently show that search for meaning positively predicts presence of meaning [27, 28]. Even in individualistic cultures which tend to show negative associations between search for meaning and well-being indices, it has been found that the positive relationship between presence of meaning and life satisfaction becomes even stronger when one is actively seeking meaning in life [29]. Also, recent daily diary research has shown that when examining the relationship between search for, and presence of meaning in the level of changes within an individual, search for meaning led to more presence of meaning the next day (e.g., [30]). Thus, we could expect that an active search for meaning can lead to a higher presence of meaning regardless of cultural context, and more so in the South Korean context.

While there is a scarcity of research on the relationship between maximization and meaning in the literature, few studies have begun to examine this link. For example, two studies conducted with cross-sectional adult samples from a collectivist culture (i.e., Filipino and Chinese college students) have found that maximization was positively associated with both search for, and presence of meaning in life [31], and with overall meaning in life [18]. A recent study with Chinese adult samples further tested the causal relationship between maximization and meaning with cross-sectional, longitudinal, and experimental designs and found that maximization tendency positively predicted meaning in life consistently across methods [32]. Together, these studies suggest that the meaning-making process may function positively in the link between maximization strategies and life satisfaction. However, whether these paths would be valid in career and relationship domains and specific developmental and cultural contexts remains as an empirical question.

### Understanding maximization in a developmental and cultural context

Although maximization is typically understood as an individual trait [11], a developmental period that may be of particular interest in understanding maximization in career and relationship domains is *established adulthood*. This developmental term has been recently introduced by Mehta et al. to indicate adults from age 30 to 45 in developed countries, which is described as a distinctive period of high commitment to career and relationship [33]. Embedded in this concept, there is an expectation that adults in their 30 s to 40 s would have chosen their field of work and marriage partner by this age. There are, however, also obvious variations of established adults such as people who remain unmarried either by choice or resistance to cultural expectations [33].

Mayseless and Keren [34] argued that finding meaning is as an important developmental task in adulthood in the domains of love and work across diverse cultures. They note, however, how much an individual will invest in making such decisions in those life domains may differ by cultural expectations and significance placed on those decisions.

In South Korea, where collective, family-centric, and patriarchal traditions prevail, unmarried individuals in their 30 s and beyond often opt for a lifestyle that offers flexibility while maintaining meaningful intimacy outside their immediate family circle [28, 35–37]. Growing up in a societal framework that assigns predetermined milestones based on age, such as education, employment, marriage, and parenthood, individuals are likely to feel pressured to fit into a set schedule. However, a growing trend towards single-person households, witnessing a tenfold increase over the past three decades has led to the emergence of neologisms like “*honjok*”, a combined Korean word, by myself and tribe, and “*bihon*”, which refers to people who choose to remain single rather than marry [38]. This shift underscores a desire among Korean individuals to carve out a life path that aligns with personal values rather than societal expectations. Indeed, research by Doh and Chung [39], employing a mixed-methods approach, revealed that Korean adults in their 20 s and older who actively pursued individual meaning and fulfillment, such as a sense of purpose or belonging, reported higher levels of happiness. A study of unmarried Korean job seekers in their 30 s [40] also found that when searching for a job, they place more importance on whether the job is one that allows them to utilize their uniqueness and strengths than on the criteria of a socially desirable job, reflecting the phenomenon of wanting to give meaning to their lives even when looking for a job. Park and Lee’s [41] study of unmarried men and women in their 30 s in South Korea also found that while self-awareness did not directly affect psychological well-being, self-awareness affect psychological well-being through higher levels of meaning in life.

Thus, in the South Korean context, people may be more likely to utilize a maximization strategy because family and social expectations and influences tend to be strong in choosing one’s partner and job [35, 42–44]. This can make the individual more vulnerable in sacrificing their satisfaction with life, as they may either make a decision too swiftly or postpone their decision much longer than they want. However, if one can find meaning in the process of maximizing, it may buffer the harmful effect of using a maximization strategy on life satisfaction, and rather make it useful strategy to fulfill their values in love and work.

### Present study

In the present study, we sought to extend and clarify previous research by exploring the relationships between maximization strategy and indicators of hedonic and eudaimonic well-being in two major life domains among unmarried, established adults in South Korea. Specifically, we tested a model which explore the mediating role of a meaning-making pathway (i.e., search-to-presence of meaning in life) in the link between relationship and career maximization strategies and life satisfaction, building on prior research. In this model, we expected that relationship and career maximization strategies would be significantly associated with life satisfaction and that this path would be sequentially mediated by the search for, and presence of meaning in life.

## Methods

### Participants and procedure

Participants were recruited via a survey company based in South Korea after receiving IRB approval. Participants who voluntarily registered to the nationwide pool completed an online questionnaire and were provided monetary rewards for participating in the survey. The inclusion criteria were South Korean adults who were in their 30 s to 40 s, currently unmarried with a full-time job. Our sample (*n* = 264) consisted of 143 (54.2%) men and 121 (45.8%) women, and the mean age was 37.27 years old (*SD* = 5.46). For the age range, participants in their 30 s were 175 (66.3%) and in their 40 s were 89 (33.7%). Participants’ relationship status included 158 single (59.8%), 54 dating (20.5%), 34 seriously dating for marriage (12.9%) and 17 casual dating (6.4%). Participants’ household monthly income was reported as follows: less than $2,000 (14.8%), $2,000–4,000 (64.4%), $4,000–6,000 (15.5%), $6,000–8,000 (4.1%) and more than $8,000 (1.1%). The sample size of this study has enough statistical power as it meets the criteria of having five observations per estimated parameter [45].

### Measures

#### Relational maximization strategy

We used the alternative search subscale of the Relational Maximization Scale (RMS) [22] to measure relational maximization strategy, which consists of five items. A 7-point Likert scale (1 = *not at all*, 7 = *very much*) was used. The scale was translated into Korean and evaluated for its construct validity through exploratory factor analysis and confirmatory factor analysis [46], which confirmed that the 5-item Korean version of the alternative subscale is reliable and valid. In this study, the Cronbach alpha of the alternative search subscale was 0.80.

#### Career maximization strategy

Career maximization strategy was measured using five items of the 10-item Career Maximizing Scale (CMS) [47]. The CMS measures the degree of evaluating options in pursuit of career decisions, and we selected five items measuring alternative search in the CMS to capture career maximization strategy. The CMS uses a five-point Likert scale ranging from *strongly agree* to *strongly disagree*. Because the CMS was not validated in Korean yet, the items were independently translated from English to Korean and the forward translation was modified. English-Korean bilingual back-translated the translated items into English and verified their equivalence with the original items (see Additional file 1 for the Korean translation of CMS). The original scale shows good internal consistency ($\alpha$=0.88) [7], and the Cronbach alpha for the Korean translation was 0.87 in this study.

#### Search for and presence of meaning

The Korean version of the Meaning in Life Questionnaire (K-MLQ) [48] which translated and validated the original MLQ [49] was used to measure the presence and search for meaning in life. The presence and search subscales consist of five items each and items were rated on a 7-point Likert-type scale ranging from 1 (*absolutely untrue*) to 7 (*absolutely true*). The reliability of each subscale in Won et al. [50] was reported as both 0.88 with a Korean college student sample [48]. In this study, the reliability of each subscale was reported as 0.94 and 0.91 for search for meaning and presence of meaning, respectively.

#### Life satisfaction

In this study, Lim’s Korean version of the Satisfaction with the Life Scale (K-SWLS) was used to measure life satisfaction, which confirmed to be a valid and reliable measurement [51]. Items are rated on a 7-point Likert-type scale ranging from 1 (*strongly disagree*) to 7 (*strongly agree*). High scores indicate a higher life satisfaction. The reliability of the K-SWLS was tested with diverse groups in South Korea which ranged from 0.77 to 0.90 [51]. In this study, the Cronbach’s alpha of the K-SWLS was 0.93.

### Data analytic plan

We tested the hypothesized model following the two-step procedure of structural equation modeling [52] using several recommended goodness-of-fit measures (e.g., χ^2^, CFI, NFI, RMSEA) to evaluate how well the hypothesized model fit the observed data [48]. In the first step, confirmatory factor analysis was conducted to test whether the measurement model had an acceptable fit. After confirming the measurement model, the structural model of the hypothesized model was tested. Additionally, a bootstrapping procedure was conducted to test the generalizability and indirect effects in the hypothesized model.

## Results

### Preliminary analyses

Prior to the main analyses, we conducted preliminary analyses and deleted five outliers which were identified using Mahalanobis distance. Our sample (*n* = 264) data were univariate and multivariate normal, thereby meeting SEM assumptions. Pearson correlations revealed positive significant associations ranging from 0.15 to 0.68 for relational maximization strategy, career maximization strategy, search for meaning, presence of meaning, and life satisfaction (see Table 1). Significant correlations were below 0.85, so multicollinearity is likely not a problem [53].

**Table 1:** Zero-order Pearson correlation among variables (*n* = 264)

	**1**	**2**	**3**	**4**	**5**
1. Relational maximization					
2. Career maximization	.26^**^				
3. Search for meaning	.26^**^	.45^**^			
4. Presence of meaning	.23^*^	.38^**^	.68^**^		
5. Life satisfaction	.23^**^	.15^*^	.34^**^	.46^**^	
* M*	4.27	3.43	4.82	4.28	3.57
* SD*	.94	.56	1.07	1.04	1.28

^**^p < .01, ^*^p < .05

### Measurement model

The proposed mediation model followed the two-step procedure. In the first step, confirmatory factor analysis was conducted to develop a measurement model with an acceptable fit. The confirmatory model consisted of five latent variables and 19 observed variables. For relationship and career maximization strategy, we used the item-to-construct balance method to create parcels [54]. For search for meaning, presence of meaning, and life satisfaction, we used each item as indicators. Observed variables were examined for whether their path coefficient from the latent variables was below 0.5 to see if latent variables were well measured by their indicators [55]. The last item in the presence of meaning was deleted from the presence of meaning latent variable because the path coefficient was below 0.5 (0.33). The measurement model without the item in the presence of meaning resulted an adequate fit, χ^2^ (125) = 269.72 (*p* < 0.001), CFI = 0.96, TLI = 0.95, RMSEA = 0.07, SRMR = 0.05. The factor analysis loading ranged from 0.75 to 0.91, providing evidence for validity of the Korean translations of the measures. Because the revised model was a better fit to the data, it was used as the baseline model for testing the structural model.

### Structural model

**Fig. 1:**
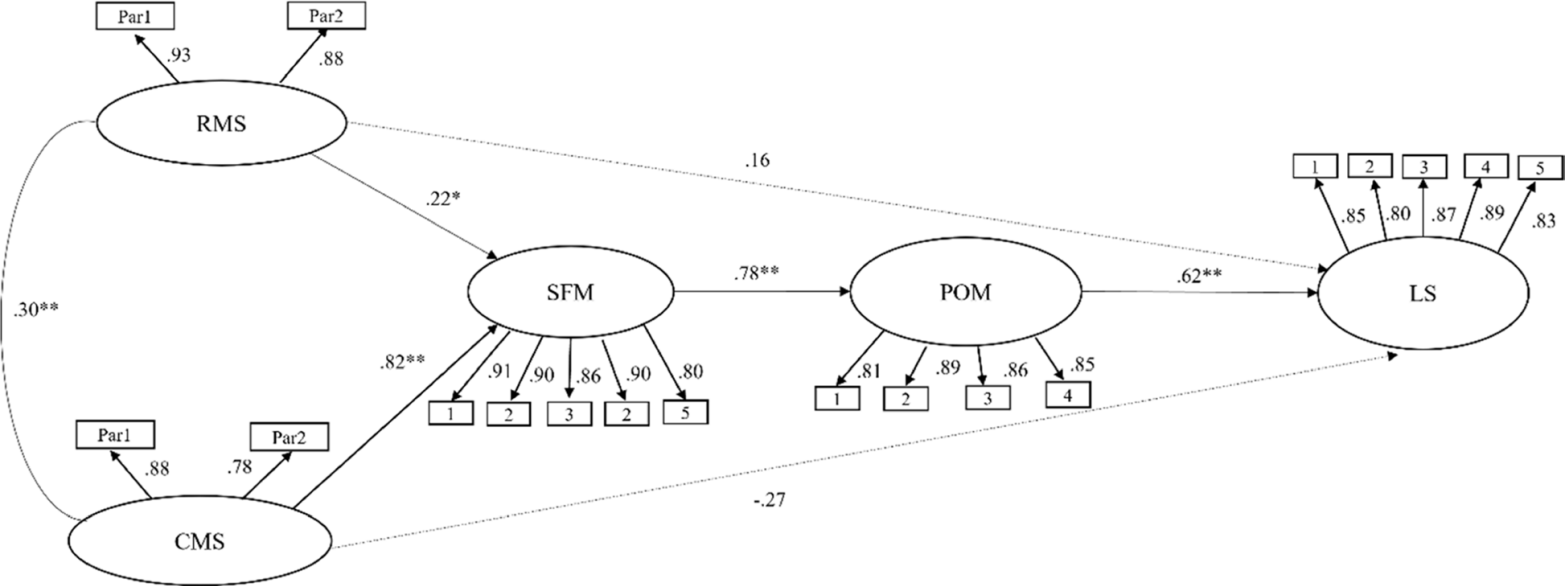
Path estimates of the structural model (*n* = 264). Note. RMS = Relational Maximization Strategy; CMS = Career Maximization Strategy; SFM = Search for Meaning; POM = Presence of Meaning; LS = Life Satisfaction; **p < .01, *p < .05

Since the measurement model was supported, we tested the fit of the structural model of the hypothesized model. As shown in Fig. 1, the results indicated that the serial mediation model had good fit to the data, *χ*^*2*^ (128) = 273.93 (*p* < 0.001), CFI = 0.96, TLI = 0.95, RMSEA = 0.07, SRMR = 0.05, AIC = 395.93. All paths except the direct paths from both relational and career maximization strategy to life satisfaction were significant.

Bootstrapping was used to assess the significance of mediated effects in multiple mediator models, allowing for the estimation of both the overall indirect effect and the significance of each individual indirect effect [2]. To test the indirect effects of search for meaning and presence of meaning, bootstrap resampling (*N* = 5,000) was used. Bootstrapping results indicated that both indirect paths were significant (Table 2). As recommended by MacKinnon & Willams [56], we reported the confidence intervals in Table 2. Given that these indirect effects were significant, the results supported a serial mediation effect via a meaning-making process (i.e., search for meaning and presence of meaning) between both relational/career maximization strategies on life satisfaction.

**Table 2:** Bootstrap estimates of the indirect effects

**Path**	**Effect**	***SE***	**95% CI** **(Lower, Upper)**
RMS → SFM → POM → LS	.16^*^	.04	.01, -.16
CMS → SFM → POM → LS	.26^**^	.04	.11, -.26

*RMS* Relational Maximization Strategy, *CMS* Career Maximization Strategy, *SFM* Search for Meaning, *POM* Presence of Meaning, *LS* Life Satisfaction

^**^p < .01, ^*^ *p* < .05

## Discussion

This study aimed to explore the relationship between maximization strategy and well-being in two important life decision domains (i.e., romantic relationship and career) with a sample of South Korean established adults in their 30 s to 40 s, and the mediating role of meaning-making in this relationship through structural equation modeling. Our findings have several important implications. First, by shedding light on the psychological process of meaning-making, this study allowed us to reassess the impact of maximization strategies on well-being [12]. While previous research has shown that higher levels of maximization tend to be associated with lower levels of well-being, our findings suggest that maximization can increase life satisfaction through the meaning-making process. For example, Kokkoris reported that maximizers have a strong hope for success which is positively correlated with subjective well-being [17]. Diab et al. found that maximization was not associated with lower psychological adjustment and life satisfaction [57], and Lai confirmed that maximization is not always negative [58]. Specifically, Lai found a positive correlation between maximization and several positive psychological indicators, including intrinsic motivation and self-efficacy [58]. Taken together, these findings suggest that maximizers seek optimal possibilities and are not satisfied with what they have achieved. They also expect good decision-making outcomes and work performance and may be more motivated to achieve through the process of finding and maintaining meaning than the satisfaction type. In other words, these findings suggest that maximizers may be more satisfied with their lives when they act proactively with positive hopes for success rather than dwelling on failure through the process of finding and giving meaning to their actions in the areas of their lives that matter to them – their careers and relationships. Taken together with previous findings, the results of our study indicate the importance of the process of searching for meaning in utilizing the maximization strategy compared to mindlessly searching for alternatives, which is consistent with previous research on the beneficial effects of search for meaning (e.g., [29]). Our results also support a strong association between presence of meaning and life satisfaction, which imply the importance of ultimately finding meaning through the search for meaning. In other words, our findings show some similarity to the notion of pleasure derived from efforts to overcome difficulties.

Secondly, the results of this study further extend the research on maximization and well-being to domain-specific maximization, particularly relational and career maximization. Interestingly, the path from career maximization strategy to search for meaning was stronger than that of relational maximization strategy. These findings seem to indicate that searching for better alternatives in relationships and career might have different meanings and thus influences individuals’ well-being in different ways. Compared to relationships, career may be related to a more basic need that has more to do with making a living. Thus, career decisions are likely to be made after proper consideration, cautious evaluation of pros and cons, and prudent decision-making before taking any trivial actions privately or officially. Mostly, career or vocation-related decisions are made under an uncertain condition, so people with career maximization tendency are likely to utilize positive cognition, such as positive future orientation [37], willingness to approach career-related opportunities (e.g., proposing a new project, negotiating salary or higher-ranking position, deepening or expanding network with related professionals) confidently, which will help them to manage uncertainty [59]. In other words, career maximization tendency might be highly related to indicators of confident decision-making, such as ambiguity tolerance [36] and career decision-making self-efficacy [59]. Therefore, as suggested in our findings, it is expected that career maximizers will put more thought and care into finding meaning and making them.

We also found that the path between relationship maximization strategy and search for meaning, while not as strong as the path between career maximization strategy and search for meaning, had a statistically significant positive relationship and a positive effect on life satisfaction, mediated by search for meaning. This finding is inconsistent with previous research that reports that relationship maximizers who are open to other relationships are likely to have a low level of life satisfaction because they have to make a large investment in their decisions [20, 60]. However, our findings do not necessarily suggest that relationship maximizers are less satisfied with their lives because they have to spend more time and to deliberate decisions about their relationships. Our findings suggest that relationship maximizers may be more satisfied with their lives if they are open to other relationship alternatives but acknowledge and find meaning in their life, which emphasizes giving and receiving love, by looking more closely at their readiness for commitment in their current romantic relationships. Further research is needed to confirm that relationship maximizers, who are established adults with a variety of relationship experiences, can be more satisfied with their lives as they go through the meaning-making process of accepting their own values and dispositions about love that do not preclude the possibility of other relationships. In sum, these results imply the unique domain-specific characteristics of the maximization and well-being relationship, in which the career domain might require more active search for meaning for individuals in their 30 to 40 s to be satisfied with their life.

Finally, this finding showed that established South Korean adults are more satisfied with their lives as they struggle to make better choices in work and love despite the implicit norms inherent in Korean culture about age-specific developmental tasks. In collectivistic cultures such as South Korea, people tend to think more importantly about how they are reflected by others and define themselves through external standards [61]. In such cultural contexts, maximizing behavior in relationships and career may be strongly influenced by social norms [21], such as being expected to obtain a decent job and getting married at a certain age. Additionally, the opportunities and value of individual choice may differ by cultural context which may moderate the relationship between maximization and well-being [11]. The South Korean culture is often described as ‘relational’ in nature, which emphasizes harmony with family and close friends [62]. Because it might be harder to keep individual standards in making important life decisions under such social pressure, people who try to search for the best option for their career and romantic partner with a clear intention may be more satisfied with their life than those who compromise their decision to societal expectations. Considering that the sample of this study were unmarried established adults in their 30-40 s, they might have received social pressure to choose a good enough option rather than maximizing their decision. Thus, those who utilized a high level of maximization strategy in relationship and career may be people who pursued their own standards while resisting to conform to social pressure.

### Study limitations and directions for future research

Despite the meaningful results of the current study, several limitations need to be addressed. First, although our sample was drawn from a nationwide pool in South Korea and equally represented gender and a range of income levels, the data may not be representative of the average South Korean unmarried, working established adult. Since this was the first study examining the mediating effect of the meaning-making process in domain-specific maximization and life satisfaction, future studies should replicate the findings by testing the model with nationally representative samples, and further examine its generalizability. Second, while our sample size demonstrated enough power given conventional criteria for structural equation modeling, a larger sample size may have detected a direct effect between relational/career maximization strategy and life satisfaction given their moderate sized correlations. Power analysis in SEM is complicated, and new apps are being developed to better deal with this issue (e.g., [63, 64]). In future work, conducting an a priori power analysis will be beneficial to ensure that the sample size has enough power to detect the effect of interest. Third, although our study did not show significant group differences based on demographic variables such as gender and income level, social expectations on career and relationship decision-making may differ by these demographic factors and thus would benefit from future exploration. Fourth, we used the original RMS [20] with a sample that included both participants in initial and ongoing decision-making processes in relationships. According to a recent study on the Revised Relational Maximization Scale (RRMS) [65], the original RMS reflects a pre-choice decision-making for singles. Therefore, future studies should use the RRMS for those who are in an ongoing decision-making process despite being in committed relationships. Finally, the significant positive relations between alternative search in both romantic relationship and career domains, and life satisfaction in this study were not consistent with previous findings that reported a negative significant relationship between them (e.g., [22]). Considering that maximization can be an adaptive or non-adaptive strategy depending on the cultural context and age group, more research is needed in diverse contexts.

## Conclusions

The findings of this study add to the literature on maximization and well-being by examining the specific relationship between relational and career maximization and life satisfaction, and the role of meaning-making in the relationship and career decision-making process. These findings have practical implications for established adults in their 30 s to 40 s who are continuing to explore options for a romantic partner and career. Instead of making important life decisions in the sake of making a choice, it would be beneficial to make meaning in the decision-making process. Particularly in a culture where it is socially encouraged to make choices in marriage or occupation at an early age like South Korea, it would be necessary to help individuals utilize their maximization strategy as a positive resource.

### Supplementary Information

**Additional file 1. **Korean translation of CMS.

## Data Availability

The datasets generated and analyzed during the current study are not publicly available because the data collection process was funded by a national institute but are available from the corresponding author on reasonable request.
