# Correction: Maximization strategies in relationship and career enhances life satisfaction through meaning making among established adults in South Korea

**DOI:** 10.1186/s40359-024-01774-x

**Published:** 2024-05-28

**Authors:** Yerin Shim, Yun-Jeong Shin, Ji-yeon Lee

**Affiliations:** 1 Department of Psychology, Chungnam National University, Daejeon, South Korea; 2 Department of Education, Seoul National University, 1 Kwanak Ro, Kwanak-gu, Seoul, 08826 South Korea; 3 Graduate School of Education, Hankuk University of Foreign Studies, 107 Imun Ro, Seoul, 130-791 South Korea

**Correction to: BMC Psychology (2024) 12:214**

10.1186/s40359-024-01672-2

Following publication of the original article, it was brought to the journal’s attention that there was an error in the Authors’ contributions declaration: the comment ‘We’d like to have both Yerun Shim and Ji-yeon Lee as first authors’. had been erroneously included in the declaration. The published article has since been corrected.

