# Supplementary material for: Maximization strategies in relationship and career enhances life satisfaction through meaning making among established adults in South Korea
Source: BMC Psychol. 2024 Apr 17;12:214. doi: 10.1186/s40359-024-01672-2 (PMC11025140; doi:10.1186/s40359-024-01672-2)
Supplement: Supplementary file 1 — Additional file 1. Korean translation of CMS. [file 40359_2024_1672_MOESM1_ESM.docx]

**Additional file 1**

*Korean Translation of CMS*

| 지금까지 살아오면서 진로 및 직업 선택을 할 때 본인의 경향성을 생각해보면서 질문에 답해주시기 바랍니다. | | | | | | |
| --- | --- | --- | --- | --- | --- | --- |
| 번호 | 질문 | 매우  동의하지  않는다 | 동의하지  않는다 | 보통이다 | 동의한다 | 매우  동의한다 |
| 1 | 나는 직장을 선택하기 전에 나에게 가능한 모든 직장을 알아야만 한다. | ① | ② | ③ | ④ | ⑤ |
| 2 | 나는 나의 커리어(진로)를 극대화시킬 직장을 선택할 것이다. | ① | ② | ③ | ④ | ⑤ |
| 3 | 나는 가장 좋은 선택지가 아닌 직장에 안주하는 것을 매우 싫어하는 편이다. | ① | ② | ③ | ④ | ⑤ |
| 4 | 진로 선택지들에 대해 생각할 때, 나는 모든 가능성에 대해 상상해보려고 한다. | ① | ② | ③ | ④ | ⑤ |
| 5 | 나는 직장을 선택하기 전에 많은 선택지를 찾아본다. | ① | ② | ③ | ④ | ⑤ |
| 6 | 나는 어떤 직장을 선택하기 전에 그 장단점을 조심스럽게 따져본다. | ① | ② | ③ | ④ | ⑤ |
| 7 | 나는 진로를 선택할 때, 가장 좋은 것을 선택하려고 항상 노력한다. | ① | ② | ③ | ④ | ⑤ |
| 8 | 나는 가장 좋은 직장을 찾으려고 끊임없이 노력한다. | ① | ② | ③ | ④ | ⑤ |
| 9 | 커리어(진로)에 있어서 나는 나 자신에게 있어 매우 높은 기준을 갖고 있다. | ① | ② | ③ | ④ | ⑤ |
| 10 | 가장 좋은 일터를 찾는 것은 나에게 매우 중요하다. | ① | ② | ③ | ④ | ⑤ |

**Table 1**

The Original English Version of the Career Maximizing Scale (10)

| Number | Questions | Strongly Disagree | Disagree | Neutral | Agree | Strongly Agree |
| --- | --- | --- | --- | --- | --- | --- |
| 1 | I need to know as much as I can about all jobs before choosing mine. | ① | ② | ③ | ④ | ⑤ |
| 2 | I will choose a job that can maximize my career. | ① | ② | ③ | ④ | ⑤ |
| 3 | I would hate to settle down for a job that is not the best choice. | ① | ② | ③ | ④ | ⑤ |
| 4 | When thinking of my career choices I try to imagine all possibilities. | ① | ② | ③ | ④ | ⑤ |
| 5 | I look up many job options before choosing my job. | ① | ② | ③ | ④ | ⑤ |
| 6 | Before agreeing to a job I carefully go over the pros and cons. | ① | ② | ③ | ④ | ⑤ |
| 7 | When choosing a career, I always put my effort into choosing the best. | ① | ② | ③ | ④ | ⑤ |
| 8 | I constantly try hard to find the best job. | ① | ② | ③ | ④ | ⑤ |
| 9 | I have very high standards for myself in career. | ① | ② | ③ | ④ | ⑤ |
| 10 | Finding the best workplace is very important for me | ① | ② | ③ | ④ | ⑤ |
